# Evaluation of sub-acute changes in cardiac function after cisplatin-based combination chemotherapy for testicular cancer

**DOI:** 10.1038/sj.bjc.6605095

**Published:** 2009-05-19

**Authors:** R Altena, E C de Haas, J Nuver, C A J Brouwer, M P van den Berg, A J Smit, A Postma, D Th Sleijfer, J A Gietema

**Affiliations:** 1 Department of Medical Oncology, University Medical Center Groningen and University of Groningen, P.O. Box 30.001, Groningen 9700 RB, The Netherlands; 2 Department of Paediatric Oncology, University Medical Center Groningen and University of Groningen, P.O. Box 30.001, Groningen 9700 RB, The Netherlands; 3 Department of Cardiology, University Medical Center Groningen and University of Groningen, P.O. Box 30.001, Groningen 9700 RB, The Netherlands; 4 Department of Internal Medicine, University Medical Center Groningen and University of Groningen, P.O. Box 30.001, Groningen 9700 RB, The Netherlands

**Keywords:** testicular cancer, cardiovascular toxicity, echocardiography, prospective

## Abstract

Long-term cardiovascular morbidity is increasingly observed in chemotherapy-treated testicular cancer survivors, but little is known of early sub-clinical changes in cardiac function. We prospectively evaluated cardiac function in testicular cancer patients by echocardiography. Systolic (Wall Motion Score Index) and diastolic (E/A-ratio and Tissue Velocity Imaging (TVI)) parameters, and serum levels of N-Terminal pro-Brain Natriuretic Peptide (NT-proBNP) were assessed before the start of chemotherapy and 1 year later. Echocardiography data were compared with an age-matched group of healthy controls. Forty-two patients treated with bleomycin, etoposide and cisplatin were evaluated (median age 27 years, range 18–50). Systolic function and E/A-ratio did not change, whereas the median TVI decreased (12.0 *vs* 10.0 cms^−1^; *P*=0.002). Median levels of NT-proBNP increased (5 *vs* 18 pmoll^−1^, *P*=0.034). Compared with controls, TVI before the start of chemotherapy was not significantly different. In conclusion, we found that at a median of 10 months after cisplatin-based treatment for testicular cancer, TVI decreased significantly, indicating a deterioration of diastolic cardiac function. Serum levels of NT-proBNP increased. The prognostic significance of these changes for future cardiovascular morbidity is not clear.

Testicular cancer (TC), the most frequent type of solid malignancy in young male adults, has become a highly curable disease since the late 1970s with the introduction of cisplatin-containing chemotherapy regimens. In the growing population of TC survivors, the occurrence of long-term treatment-induced organ damage is increasingly recognised as an important cause of morbidity (Feldman *et al*, 2008). Compared with the general population, long-term TC survivors have a higher incidence of second malignant neoplasms and cardiovascular disease (Meinardi *et al*, 2000; Strumberg *et al*, 2002; Huddart *et al*, 2003; van den Belt-Dusebout *et al*, 2007).

In earlier studies, investigating cardiac morbidity in long-term TC survivors at a median of 7–14 years after chemotherapy, we found diastolic dysfunction in 17–33% of patients (Meinardi *et al*, 2000; Nuver *et al*, 2005a). Sub-clinical signs of vascular toxicity were found in a prospective study in TC patients 10 weeks after cisplatin-based chemotherapy (Nuver *et al*, 2005b). Still, little is known about (sub-) acute cardiotoxicity in TC patients, and to our knowledge, no studies report on early cardiotoxicity. Furthermore, it is not established which parameters are useful in the early assessment of cardiac damage.

In addition to obtaining insight in the extent and timing of cardiac complications of chemotherapeutic treatment for TC, evaluation of parameters for early (sub-clinical) cardiac dysfunction may enable the identification of patients who are at risk for future cardiovascular events. Echocardiography is a convenient and frequently used method to assess cardiac function, enabling evaluation of both systolic and diastolic function parameters. Biochemical markers can also be used to evaluate cardiac status. N-Terminal pro-Brain Natriuretic Peptide (NT-proBNP) is produced by ventricular cells in response to increased mechanical load and wall stretch. Plasma levels are used as a prognostic indicator in different stages and causes of cardiac disease (Doust *et al*, 2005). However, its role in detecting chemotherapy-induced cardiac morbidity has not been established yet (Bryant *et al*, 2007; Ekstein *et al*, 2007).

In this prospective cohort study, we investigated echocardiographic and biochemical changes before and at 1 year after the start of cisplatin-containing chemotherapy for disseminated TC.

## Patients and methods

### Patients

All consecutive patients with disseminated TC, scheduled to receive cisplatin-containing chemotherapy as first-line therapy at the University Medical Centre Groningen, the Netherlands, between December 2000 and October 2004 were asked to participate in a study investigating the chemotherapy-induced acute cardiovascular toxicity (Nuver *et al*, 2005b). Exclusion criteria were extra-pulmonary visceral metastases, earlier radiotherapy, pre-treatment history of cardiac disease, use of erythropoietin and an age older than 55 years at the start of chemotherapy. The study was approved by the local ethics committee and written informed consent was obtained from all participants.

After orchidectomy, all patients received three or four three-weekly courses of combination chemotherapy consisting of bleomycin (30 mg on days 2, 8 and 15), etoposide (100 mg m^–2^ on days 1–5 of each course) and cisplatin (20 mg m^–2^ on days 1–5 of each course). Patients were admitted to the hospital for hydration with 3 l of 0.9% NaCL per day during the first 6 days of each course. All patients received dexamethason and odansetron as standard anti-emetic therapy.

Reference data were obtained from healthy male siblings of adult childhood cancer survivors, who had participated as control subjects in a cross-sectional study on late cardiovascular sequelae of treatment for childhood cancer. Out of these healthy male siblings, a control group was selected with a comparable median age as the TC patients. Measurements in the controls were carried out under similar circumstances and methods.

### Measurements

Measurements were done within 1 week before the start of chemotherapy and ∼1 year after the completion of treatment.

### Echocardiography

Echocardiography was carried out by a skilled technician at the same laboratory using conventional equipment (General Electrical VIVID 7 system, Horton, Norway, with a 2.5 MHz probe) and consisted of two-dimensional echocardiography, colour-flow mapping and, since 2002, tissue velocity imaging (TVI) (Cheitlin *et al*, 2003). Left ventricular end-diastolic dimension (LVEDD, normal 36–54 mm), left ventricular end-systolic dimension (LVESD, normal 23–40 mm), posterior and septal wall thickness (normal 7–11 mm) were measured on M-mode recordings obtained in the standard left ventricular parasternal long-axis view. The parasternal, transverse and longitudinal dimensions of the left atrium were attained.

For the analysis of systolic function, the left ventricle was divided into 16 segments. Each segment was visually scored between 1 and 4 (1=normokinesia, 2=hypokinesia, 3=akinesia, 4=dyskinesia). The wall motion score index (WMSI) was the mean score for all the analysed segments. A WMSI of 1.00 was considered normal.

Diastolic function measurements included the mitral valve inflow velocities in early (E) and late (atrial; A) diastole (E/A-ratio, normal >1.00) and tissue velocity imaging of early diastole (TVI Et). Tissue velocity imaging of early diastole was the mean of measurements at the septal, lateral, inferior and anterior mitral annulus (normal >8.0 cm s^–1^; decreases indicate deterioration of diastolic function). In addition E/E′ was calculated from the peak E velocity and the mean TVI Et (normal <15; increases reflect declines in diastolic function; E/E′ >15 is considered as diastolic dysfunction). E/E′ is currently regarded as a sensitive method for assessing diastolic heart failure (Paulus *et al*, 2007).

### NT-proBNP

N-Terminal pro-Brain Natriuretic Peptide (lower limit of detection 5.0 pmol l^–1^; normal value <14.75 pmol l^–1^) was measured in plasma using an immunoassay (Roche Diagnostics, Mannheim, Germany) from samples that were drawn concomitant with the echocardiographic recordings.

### Cardiovascular risk factors (CRFs)

Cardiovascular risk factors were estimated before the start of chemotherapy. Hypercholesterolaemia was defined as a fasting level of cholesterol >6.5 mmol l^–1^; diabetes mellitus as a fasting level of glucose >7.0 mmol l^–1^. Obesity as body mass index (BMI) >27.8 kg m^–2^. Blood pressure (BP) was estimated as a single recording on one arm in supine position in a quiet room after a minimal rest period of 10 min. Furthermore, an ambulatory BP device (Spacelab 90207; Spacelabs Inc., Redmond, WA, USA) was used to document BP every 30 min during a 24h period. The control subjects had a single BP recording.

The criteria for hypertension were defined as a mean 24h BP >135/85 mm Hg, a single BP >145/95 mm Hg and/or the use of anti-hypertensive medication.

### Statistics

Statistical analyses were carried out in the statistical software package SPSS for Windows version 14.0 (SPSS Inc., Chicago, IL, USA). For comparisons the *χ*^2^-test and the non-parametric Mann–Whitney test were used. To calculate changes within a patient the Wilcoxon signed-rank test was used on the paired samples in those patients where both variables were available. Regressions were calculated with Spearman's correlation. Two-sided *P*-values ⩽0.05 were considered to indicate significant differences.

*Post-hoc* power analysis showed that, with a power of 80% and a two-sided significance level (alpha) of *P*<0.05, a 3.3% change in TVI Et could be detected in 19 patients, with a correlation coefficient between two measurements under equal circumstances of ⩾0.75 and variation-coefficient for TVI Et of 7.1% (Kowalski *et al*, 2001).

## Results

### Patients

Between December 2000 and October 2004, 65 TC patients were enrolled in the study (Nuver *et al*, 2005b). In 54 of these 65 patients an echocardiogram was performed before the start of chemotherapy. Twelve out of 54 (22%) patients received no echocardiogram after the completion of cisplatin-based chemotherapy, because of the following reasons: death (*n*=1), progressive disease (*n*=1) or logistic reasons (data not evaluable *n*=3; moved out of referral area *n*=1; not performed *n*=6). Therefore, 42 patients underwent an echocardiogram before and within 1 year after the completion of cancer treatment. The baseline characteristics like age, disease status and echocardiographic parameters of the patients (*n*=12) who did not receive an echocardiogram after treatment were not different from the patients that completed two echocardiographic evaluations (data not shown).

Baseline characteristics of the 42 patients with two echocardiographic evaluations are shown in Table 1. Of the 42 evaluable patients, 12 had pulmonary metastases and one underwent thoracotomy for resection of pulmonary lesions after the completion of chemotherapy.

The control group consisted of 42 healthy males, whose median age was 28 years (Table 2); none of them was known to have co-morbidity.

### Echocardiography

#### Echocardiography before treatment

Before the start of treatment, one out of 33 patients had a WMSI >1.00 (3.0%). Four out of 41 patients had abnormal wall motion (all local hypokinesia; 9.8%). Three out of 42 had valve dysfunction (pulmonal (*n*=2) and tricuspid valve insufficiency (*n*=1); 7.1%).

The E/A-ratio was <1.00 in 8 out of 42 patients (19.0%); 1 out of 22 assessed patients had a TVI Et <8.0 cm s^–1^ (4.5%). None of these 22 patients had an E/E′ >15. Median values and ranges are summarised in Tables 2 and 3.

Of the controls, 1 out of 42 had had a WMSI >1.00. One out of 42 had an E/A radion <1.00; 1 out of 42 had a TVI Et mean <8.00 cm s^–1^. For median values and ranges, see Table 2.

The median WMSI and TVI Et in patients and the controls was not different (*P*=0.259; *P*=0.713), whereas the median E/A-ratio was higher in the controls (*P*<0.0001, Table 2).

#### Echocardiography after treatment

At a median of 10 months after the completion of treatment, 4 out of 28 patients had a WMSI >1.00 (14.3%). Seven out of 40 patients had wall motion abnormalities (17.5%, local and/or diffuse hypo- and akinesia).

The E/A-ratio was <1.00 in 7 out of 41 patients (17.1%); 2 out of 25 assessed patients had a TVI Et <8 cm s^–1^ (8.0%). None of the 25 patients had an E/E′ >15.

#### Changes in echocardiography before and after treatment

Cardiac dimensions and the median WMSI did not change during the year after treatment (Table 3). The percentage of patients with wall motion abnormalities did not change (9.8 *vs* 17.5%; *P*=0.309), whereas the number of patients with a WMSI >1.00 increased (*P*=0.01). The seven patients with post-treatment wall motion abnormalities included the four patients with pre-treatment abnormalities; all of them had deterioration of wall motion abnormalities. Two of them developed pulmonary embolisms during the treatment (Nuver *et al*, 2005b). Three patients newly developed wall motion abnormalities at 1 year after treatment; one had a myocardial infarction during chemotherapy.

**Figure 1:**
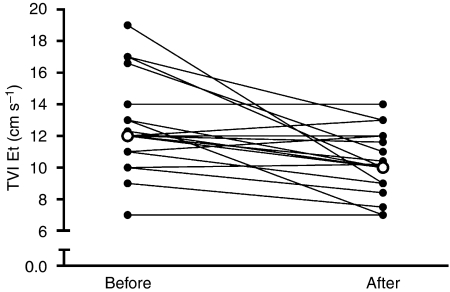
The tissue velocity imaging of early diastole (TVI Et) in 19 patients before and after chemotherapy; open circles represent median values. Median TVI Et before treatment 12.0 cm s^–1^ (range 7–19), after treatment 10.0 cm s^–1^ (range 7–17); *P*=0.002.

Paired observations of the TVI Et were available in 19 TC patients. The median TVI Et decreased significantly after treatment (*P*=0.002; Figure 1); the median E/A-ratio did not change (see Table 3). In addition, the median E/E′ increased (*P*=0.002; Table 3).

Age correlated with pre-treatment E/A-ratio (*R*=−0.50; *P*=0.001), but not with pre-treatment TVI Et (*R*=−0.27; *P*=0.219) or Δ TVI Et (difference between pre- and post-treatment TVI Et; *R*=0.21; *P*=0.382).

### NT pro-BNP

**Figure 2:**
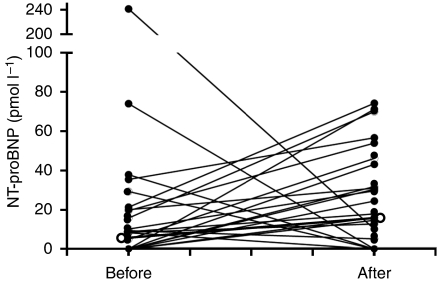
Serum levels of N-Terminal pro-Brain Natriuretic Peptide (NT-proBNP) in 32 patients before and after treatment (normal ⩽14.75 pmol l^–1^); open circles represent median values. Median NT-proBNP before treatment 5 pmol l^–1^ (range <5–242); after treatment 18 pmol l^–1^ (range <5–114); *P*=0.034.

The median level of NT-proBNP increased significantly (*n*=32; pretreatment 5 pmol l^–1^ (range <5–242); post-treatment 18 pmol l^–1^ (range <5–114); *P*=0.034, Figure 2). One extreme pre-treatment value (242 pmol l^–1^) was in a patient with a TVI Et of 7.0 cm s^–1^ before treatment.

Levels of NT-proBNP and Δ NT-proBNP did not correlate with the echocardiographic parameters before and after treatment, and neither correlated with changes in systolic and/or diastolic parameters.

### Cardiovascular risk factors

Before treatment, CRFs of the 42 patients consisted of obesity (*n*=6), smoking (*n*=14 current smokers; *n*=5 former smokers) and hypercholesterolaemia (*n*=1). The median BMI of the TC patients was 24.2 kg m^–2^ (see Table 2). Nine out of 42 patients (21%) had hypertension on 24h ambulatory BP recordings; 1 out of 42 patients was on anti-hypertensive medication (*β*-blocker). None had diabetes mellitus.

Seven out of 42 control subjects were obese; the median BMI was 25.0 kg m^–2^ (Table 2), which was not different from the TC patients (*P*=0.656). The systolic BP was significantly higher in the patients (*P*<0.0001); the diastolic BP was not different (*P*=0.237).

At 1 year after treatment, CRF consisted of obesity (*n*=8), smoking (current smokers *n*=11) and hypercholesterolaemia (*n*=3). Four out of 38 patients (11%) had hypertension on 24h BP recordings.

Both systolic and diastolic BP decreased after treatment compared with baseline (see Table 3). The pre-treatment systolic BP correlated with Δ TVI (*R*=0.67, *P*=0.006) and the TVI Et at baseline (*R*=0.63, *P*=0.002). E/A-ratio and Δ E/A-ratio did not correlate with both systolic and diastolic BP. The E/A-ratio in TC patients before treatment correlated with the BMI before treatment (*R*=−0.49, *P*=0.001), which was also true for pre-treatment TVI Et and BMI (*R*=−0.50; *P*=0.019). The WMSI before treatment did not correlate with the BMI.

Smokers had a lower E/A-ratio before treatment (E/A-ratio of smokers 1.31 (range 0.74–2.42) *vs* non-smokers 1.56 (0.87–2.41); *P*=0.039). The TVI Et as well as other echocardiographic parameters, BP and levels of NT-proBNP were not different between smokers and non-smokers.

### Events

Nuver *et al* described acute cardiovascular events in this patient group (Nuver *et al*, 2005b). During treatment, two patients had a myocardial infarction and three had pulmonary embolisms. Omission of patients with cardiovascular events from the analyses did not change the results significantly (data not shown).

## Discussion

In this group of TC patients, we observed changes in TVI Et and E/E′ within 1 year after cisplatin-based chemotherapy, representing a deterioration of diastolic cardiac function. Furthermore, serum levels of NT-proBNP increased.

Several authors report changes in cardiovascular status within years to decades after chemotherapeutic treatment for TC (Meinardi *et al*, 2000; Strumberg *et al*, 2002; Huddart *et al*, 2003; van den Belt-Dusebout *et al*, 2007), but little is known of the early changes in cardiac function in these patients. Regarding treatment-related cardiotoxicity from various cancer treatments, it was recently postulated that diastolic cardiac function deteriorates before the development of systolic dysfunction (Ewer and Lenihan, 2008). In left ventricular dysfunction of various origins, a deterioration of diastolic function can be present in the absence of systolic impairment (Lester *et al*, 2008), and sub-clinical diastolic dysfunction frequently precedes a drop in systolic parameters (Zile and Brutsaert, 2002).

Echocardiography is a frequently used method for assessing cardiac function, which has the advantage that it enables a reliable estimation of diastolic function by means of more recently introduced parameters, such as TVI Et and E/E′. Other diastolic parameters, like the E/A-ratio, are largely dependent on preload conditions (Hurrell *et al*, 1997; Sohn *et al*, 1997), resulting in significant intra-individual variation.

The TVI Et assesses the velocity of the myocardium at different angles from the mitral valve, instead of blood-flow velocities, and is therefore independent of loading conditions (Sohn *et al*, 1997; Nikitin and Witte, 2004), resulting in less intra-individual variation. This parameter is considered an important and reliable early predictor for the development cardiac dysfunction in other causes of cardiac disease (Nikitin and Witte, 2004). From several small studies in adult childhood cancer survivors, it seemed to be a valuable parameter in defining diastolic (dys-) function (Kapusta *et al*, 2001; Brouwer *et al*, 2006; Tassan-Mangina *et al*, 2006; Galderisi *et al*, 2007). E/E′ is a derivative of TVI Et and the E velocity, thereby including the end-diastolic left ventricular filling pressure in addition to myocardial velocities. This parameter is currently regarded as a valuable non-invasive method for diagnosing diastolic heart failure (Paulus *et al*, 2007). Declines in diastolic cardiac function are reflected by increases in E/E′ as well as decreases in TVI Et.

The pre-treatment echocardiography parameters in our TC patient group corresponded with the same measurements in an age-matched group of healthy controls. In the patient group, a correlation existed between age and pre-treatment E/A-ratio, but not between age and TVI Et or Δ TVI Et. This is relevant, as ageing is associated with a physiological decline in diastolic cardiac function (Galderisi, 2005). An age-dependent decrease in TVI Et has been shown in healthy controls, but this has only been evaluated in cohorts with larger differences in age and with longer time intervals (Wierzbowska-Drabik *et al*, 2008).

Several CRFs can lead to declines in diastolic function, including hypertension and obesity (Zile and Brutsaert, 2002). In this study, the pre-treatment systolic BP correlated with Δ TVI Et. Contrarily, the BMI did not correlate with the changes in TVI Et. The significant decrease in systolic and diastolic BP on the 24h ambulatory recordings was also found in the larger study investigating chemotherapy-induced acute cardiovascular toxicity (Nuver *et al*, 2005b). The causes for this finding are not known, but may at least partly be attributed to a relatively high BP before the start of treatment, which is confirmed by the finding of a higher systolic BP in the patients compared with the age-matched controls. This high BP might be because of stress before the initiation of treatment.

In this study we did not further investigate explanations for this cardiovascular toxicity. The main causes are thought to be related to direct damage to cardiomyocytes and/or the extracellular matrix, as well as sub-clinical vascular injury that induces endothelial dysfunction. Furthermore, the presence of pre-treatment elevations in BP may have resulted in impaired relaxation of the left ventricle, thereby leading to diastolic function decline. Of note, the increase in serum NT-proBNP levels is in accordance with the picture of cardiac damage.

It is unknown whether a deterioration of diastolic cardiac function during the first year after chemotherapy for TC will progress to clinically relevant cardiac disease. It can be hypothesised that the hearts of these relatively young patients can compensate for the chemotherapy-induced damage. On the contrary, they are at increased risk of developing an unfavourable cardiovascular-risk profile (Meinardi *et al*, 2000; Strumberg *et al*, 2002; Nuver *et al*, 2005c; Haugnes *et al*, 2007), which can contribute to the development of long-term cardiac failure.

In conclusion, we observed significant changes in TVI Et and E/E′ within 1 year after cisplatin-based treatment for TC, indicating a deterioration of diastolic cardiac function. The prognostic significance of this disturbed diastolic function after chemotherapy for future cardiovascular morbidity is not clear, but it might eventually lead to overt cardiac morbidity. Further longitudinal research in TC survivors is needed to obtain more insight in sub-clinical changes in cardiac function.

## Figures and Tables

**Table 1:** Characteristics of participating patients with testicular cancer

Number of patients	42
	
*Age at the start of treatment*	
Median (range)	27 (18–50) years
	
*Interval between echocardiography*	
Median (range)	10 (6–15) months
	
*Diagnosis (n)*	
Non-seminoma	40
Extra-gonadal non-seminoma	1
Seminoma	1
	
*Stage of disease(n)* a	
Stage II	28
Stage III	3
Stage IV	11
	
*Prognosis category (n)* b	
Good	28
Intermediate	14
Poor	0
	
*Treatment (n)*	
3 or 4 × BEP^c^	41
2 × BEP, 1 × VIP^d^	1

a Royal Marsden classification.

b According to the International Germ Cell Cancer Collaborative Group (IGCCCG).

c BEP, bleomycin, etoposide, cisplatin; the number of cycles was on the basis of the prognostic classification.

d VIP, etoposide, ifosfamide, cisplatin.

**Table 2:** Baseline characteristics and cardiac parameters of testicular cancer patients and healthy control subjects

	**Patients**		**Controls**		
	***n* a**	**Median (range)**	***n***	**Median (range)**	***P*-value^b^**
Age	42	27 (18–50)	42	28 (18–42)	0.907
Systolic blood pressure	30	133 (110–180)	42	115 (98–156)	<0.0001
Diastolic blood pressure	30	80 (50–130)	42	79 (60–96)	0.237
Body mass index	30	24 (19–38)	42	25 (19–31)	0.656
TVI Et	22	12.0 (7–19)	42	12.4 (9–15)	0.713
E/A ratio	42	1.38 (0.73–2.84)	42	1.69 (1.02–2.66)	<0.0001
WMSI	33	1.00 (1.00–1.23)	42	1.00 (1.00)	0.259

a Number of patients in which the respective parameters were available.

b Mann–Whitney test.

**Table 3:** Echocardiographic parameters of testicular cancer patients before and after chemotherapeutic treatment

	**Before**		**After**		
	***n* a**	**Median (range)**	***n***	**Median (range)**	***P*-value^b^**
*Systolic parameters*					
WMSI	33	1.00 (1.00–1.23)	28	1.00 (0.81–1.44)	0.273
					
*Structural dimensions*					
Septum (mm)	42	10 (6–12)	41	10 (7–14)	0.427
Posterior wall (mm)	41	10 (8–12)	41	9 (7–11)	0.309
LVEDD (mm)	42	50 (41–57)	41	50 (41–55)	0.861
LVESD (mm)	41	32 (18–42)	41	32 (21–41)	0.741
LA parasternal (mm)	42	35 (25–42)	41	35 (25–46)	0.198
LA length (mm)	40	57 (44–69)	41	58 (46–69)	0.764
LA transverse (mm)	24	38 (29–45)	31	39 (29–52)	0.267
Heartbeat (bpm)	34	70 (48–105)	31	66 (48–105)	0.235
Valve dysfunction (*n*)	42	3 (7.1%)	42	5 (11.9%)	0.457
					
*Diastolic parameters*					
Peak E velocity (cm s^–1^)	42	0.76 (0.58–1.06)	41	0.82 (0.58–1.31)	0.302
Peak A velocity (cm s^–1^)	42	0.59 (0.31–0.99)	41	0.61 (0.35–1.38)	0.097
E/A-ratio	42	1.38 (0.73–2.84)	41	1.35 (0.53–2.30)	0.259
TVI Et (cm s^–1^)^c^	22	12.0 (7–19)	25	10.0 (7–17)	0.002
E/E′^c^	22	6.2 (3.8–12.4)	25	7.6 (4.1–13.1)	0.002
					
*Blood pressure* d					
Systolic (mm Hg)	42	129 (107–154)	38	123 (106–153)	0.011
Diastolic (mm Hg)	42	74 (58–99)	38	70 (54–106)	0.018

a Number of patients in which the respective parameters were available.

b Wilcoxon signed-rank test.

c 19 paired observations available.

d 24 h ambulatory blood pressure recordings.
